# 5mC DNA methylation modification-mediated regulation in tissue functional differentiation and important flavor substance synthesis of tea plant (*Camellia sinensis* L.)

**DOI:** 10.1093/hr/uhad126

**Published:** 2023-06-13

**Authors:** Weilong Kong, Qiufang Zhu, Qing Zhang, Yiwang Zhu, Jingjing Yang, Kun Chai, Wenlong Lei, Mengwei Jiang, Shengcheng Zhang, Jinke Lin, Xingtan Zhang

**Affiliations:** National Key Laboratory for Tropical Crop Breeding, Shenzhen Branch, Guangdong Laboratory for Lingnan Modern Agriculture, Genome Analysis Laboratory of the Ministry of Agriculture, Agricultural Genomics Institute at Shenzhen, Chinese Academy of Agricultural Sciences, Shenzhen, Guangzhou 518120, China; College of Horticulture, Fujian Agriculture and Forestry University, Fuzhou, Fujian 350002, China; National Key Laboratory for Tropical Crop Breeding, Shenzhen Branch, Guangdong Laboratory for Lingnan Modern Agriculture, Genome Analysis Laboratory of the Ministry of Agriculture, Agricultural Genomics Institute at Shenzhen, Chinese Academy of Agricultural Sciences, Shenzhen, Guangzhou 518120, China; National Key Laboratory for Tropical Crop Breeding, Shenzhen Branch, Guangdong Laboratory for Lingnan Modern Agriculture, Genome Analysis Laboratory of the Ministry of Agriculture, Agricultural Genomics Institute at Shenzhen, Chinese Academy of Agricultural Sciences, Shenzhen, Guangzhou 518120, China; National Key Laboratory for Tropical Crop Breeding, Shenzhen Branch, Guangdong Laboratory for Lingnan Modern Agriculture, Genome Analysis Laboratory of the Ministry of Agriculture, Agricultural Genomics Institute at Shenzhen, Chinese Academy of Agricultural Sciences, Shenzhen, Guangzhou 518120, China; National Key Laboratory for Tropical Crop Breeding, Shenzhen Branch, Guangdong Laboratory for Lingnan Modern Agriculture, Genome Analysis Laboratory of the Ministry of Agriculture, Agricultural Genomics Institute at Shenzhen, Chinese Academy of Agricultural Sciences, Shenzhen, Guangzhou 518120, China; National Key Laboratory for Tropical Crop Breeding, Shenzhen Branch, Guangdong Laboratory for Lingnan Modern Agriculture, Genome Analysis Laboratory of the Ministry of Agriculture, Agricultural Genomics Institute at Shenzhen, Chinese Academy of Agricultural Sciences, Shenzhen, Guangzhou 518120, China; National Key Laboratory for Tropical Crop Breeding, Shenzhen Branch, Guangdong Laboratory for Lingnan Modern Agriculture, Genome Analysis Laboratory of the Ministry of Agriculture, Agricultural Genomics Institute at Shenzhen, Chinese Academy of Agricultural Sciences, Shenzhen, Guangzhou 518120, China; National Key Laboratory for Tropical Crop Breeding, Shenzhen Branch, Guangdong Laboratory for Lingnan Modern Agriculture, Genome Analysis Laboratory of the Ministry of Agriculture, Agricultural Genomics Institute at Shenzhen, Chinese Academy of Agricultural Sciences, Shenzhen, Guangzhou 518120, China; College of Horticulture, Fujian Agriculture and Forestry University, Fuzhou, Fujian 350002, China; National Key Laboratory for Tropical Crop Breeding, Shenzhen Branch, Guangdong Laboratory for Lingnan Modern Agriculture, Genome Analysis Laboratory of the Ministry of Agriculture, Agricultural Genomics Institute at Shenzhen, Chinese Academy of Agricultural Sciences, Shenzhen, Guangzhou 518120, China

## Abstract

In plants, 5mC DNA methylation is an important and conserved epistatic mark involving genomic stability, gene transcriptional regulation, developmental regulation, abiotic stress response, metabolite synthesis, etc. However, the roles of 5mC DNA methylation modification (5mC methylation) in tea plant growth and development (in pre-harvest processing) and flavor substance synthesis in pre- and post-harvest processing are unknown. We therefore conducted a comprehensive methylation analysis of four key pre-harvest tissues (root, leaf, flower, and fruit) and two processed leaves during oolong tea post-harvest processing. We found that differential 5mC methylation among four key tissues is closely related to tissue functional differentiation and that genes expressed tissue-specifically, responsible for tissue-specific functions, maintain relatively low 5mC methylation levels relative to non-tissue-specifically expressed genes. Importantly, hypomethylation modifications of *CsAlaDC* and *TS*/*GS* genes in roots provided the molecular basis for the dominant synthesis of theanine in roots. In addition, integration of 5mC DNA methylationomics, metabolomics, and transcriptomics of post-harvest leaves revealed that content changes in flavor metabolites during oolong tea processing were closely associated with transcription level changes in corresponding metabolite synthesis genes, and changes in transcript levels of these important synthesis genes were strictly regulated by 5mC methylation. We further report that some key genes during processing are regulated by 5mC methylation, which can effectively explain the content changes of important aroma metabolites, including α-farnesene, nerolidol, lipids, and taste substances such as catechins. Our results not only highlight the key roles of 5mC methylation in important flavor substance synthesis in pre- and post-harvest processing, but also provide epimutation-related gene targets for future improvement of tea quality or breeding of whole-tissue high-theanine varieties.

## Introduction

In plants, epigenetic regulation includes DNA methylation, RNA methylation, non-coding RNA, histone modification, nucleosome remodeling, and histone variation, which together form a complex upstream epigenetic regulatory system [1–3]. Of these elements, 5mC DNA methylation modification (hereafter abbreviated as 5mC methylation) participates in the regulation of a variety of physiological and biochemical processes, such as cell differentiation, immune responses, gene expression, and stress responses, and plays an indispensable role in plant growth and development [4]. The 5mC methylation status of plant genomic DNA undergoes increased or decreased changes under unfavorable external stresses to regulate chromatin structure, which in turn enables the regulation of expression of genes associated with responses to environmental stimuli [5–7]. Also, 5mC methylation was associated with fruit ripening in sweet orange and tomato [8, 9]. However, the tea plant (*Camellia sinensis* (L.) O. Kuntze) is a non-model crop lacking a mature genetic transformation system, and little research has been performed on the epigenetic dimensions of this important beverage plant to date. The limited findings only demonstrate that epigenetic regulators may play important regulatory roles in the cold stress response of tea plants [4, 5], abscisic acid (ABA) accumulation during withering [6], indole content changes under post-harvest damage stimulation [7], and the accumulation of volatile terpenoids during the post-harvest withering process [8] involving 5mC methylation [4, 5], 5mC methylation and histone modifications [6, 7], and m6A RNA demethylation [8]. These several previous studies revealed the critical roles of 5mC methylation in pre-harvest (cold stress) [4, 5] and post-harvest processing (ABA and indole content) [6, 7] of the tea plant, but the important roles of 5mC methylation in pre-harvest and post-harvest processing of tea plant may be seriously underestimated due to the lack of global high-throughput 5mC methylation studies. For example, the important role of 5mC methylation in animal tissue differentiation has been highlighted and the abnormal methylation profile of cancerous cell tissues has been utilized for cancer diagnosis [9–11]. Similarly, pre-harvest tissues of the tea plant are also highly differentiated so as to be responsible for their characteristic functions [12], and the key ‘freshness’ substance, theanine, is efficiently synthesized only in the roots of the tea plant [13]. Does 5mC methylation play a role in the functional differentiation of tissues and the root tissue-biased synthesis of theanine in the tea plant? In post-harvest processing, it is also unclear whether 5mC methylation is related to the synthesis of other important flavor substances, such as terpenes, catechins, and lipids, in addition to indole [7]. To fully investigate the roles of 5mC methylation in growth regulation and pre- and post-harvest flavor synthesis in the tea plant, a global high-throughput multiomics analysis including 5mC methylation sequencing has to be performed on key pre- and post-harvest samples.

There is an increasing interest in the study of the synthesis of tea quality compounds during growth (pre-harvest) [14–19] and post-harvest processing [20–24]. In pre-harvest processing, several genome-wide association study (GWAS) analyses and genome-wide linkage analyses in tea plant populations identified some catechin-, caffeine-, theanine-, or terpenoid-associated single-nucleotide polymorphisms (SNPs), genetic loci, candidate genes, and important molecular markers [14, 17–19]. However, the epistatic regulation of important flavor substances in tea plant pre-harvest tissues remained unknown. Oolong tea is subjected to various stresses during post-harvest processing, and various aroma- and flavor-related metabolites are produced in large quantities under different stress conditions, thus giving the finished tea its unique quality and health attributes [1, 25]. Previous extensive studies have been conducted on changes in water and hormone content [26], changes in secondary metabolite content [23, 27–30], changes in protein content [27, 29, 30], and changes in gene expression [21–23, 31] during post-harvest processing. In short, after picking tea leaves, spreading them for several hours to facilitate the withering process under dehydration stress results in a reduction in the herbaceous aroma and an accompanying reduction of ~5–10% in weight due to water loss from the leaves [6, 25]. During the subsequent turnover process, tea leaves continue to be subjected to multiple stresses, such as dehydration, low temperature, and mechanical damage. The mechanical damage and severe water loss of cells will cause a variety of drastic enzymatic reactions to occur, involving the synthesis and degradation of a variety of secondary metabolites [26], and a large number of aroma-related substances are formed in large quantities during the turnover process, which is also considered to be the main step in the aroma production and taste substance formation of tea leaves [7, 25, 30]. Next, the fixation process completely deactivates the entire leaf and terminates various enzymatic processes, allowing the quality-related substance components and contents accumulated during the first two processes to be fixed. Metabolomics and transcriptomics studies showed that stress-induced increases in the content levels of target metabolites (including flavor-related metabolites) during processing were mainly due to upregulation of the expression level of their metabolite synthesis genes. For example, low temperature and sustained mechanical damage during the tea leaf turnover process cooperatively increased the contents of indole, nerolidol, and jasmonolactone because stress induced upregulated expression of the *CsTSB2* (*tryptophan synthase β-subunit 2*), *CsLOX1* (*lipoxygenase*), and *CsNES* (*(E)-nerolidol synthase*) genes [7, 32–35]. However, the lack of global 5mC methylation-mediated upstream regulation for the synthesis of important flavor substances during post-harvest processing has hindered our deep understanding of this important post-harvest process, when miraculous changes in flavor occur.

Recent molecular biology experiments on the growth and post-harvest processing of the tea plant have improved our knowledge of the mechanisms of synthesis of important flavor substances, namely, terpenes, catechins, and theanine [1, 7, 13, 25, 36–43]. Substances such as terpenes originate from two precursors, isopentenyl diphosphate (IPP) and dimethylallyl pyrophosphate (DMAPP), and their biosynthetic metabolic pathways include two main ones, one of which is the mevalonate (MVA) pathway involved in the biosynthesis of sterols, sesquiterpenes, triterpenes, and so on [36]. The other one is the 2-C-methyl-d-erythritol-4-phosphate (MEP) pathway responsible for the biosynthesis of monoterpenes, diterpenes, carotenoids, etc. [37]. Among them, the key roles of nerolidol synthase (CsNES), linalool synthase (CsLIS), and α-farnesene synthase (CsAFS1) in tea plant nerolidol, linalool, and α-farnesene synthesis have been emphasized several times [1, 25, 36, 38]. Catechins are synthesized through two sequential pathways, the phenylpropanoid and flavonoid pathways, and the MBW (MYB–bHLH–WD40) complex, consisting of bHLH, MYB, and WD40 transcription factors (TFs), is thought to affect the expression levels of key genes of the catechin synthesis pathway, such as *anthocyanin reductase* (*ANR*) [39, 40]. Theanine is predominantly synthesized in tea plant roots from ethylamine and glutamate via theanine synthetase [13]. However, little is still known about the upstream regulatory mechanisms regulating the expression of these key flavor-related genes, and only a few TFs were reported to be involved in the upstream regulation of genes that form flavor-related compounds, such as CsMYC2 regulating indole accumulation [7] and CsMYB1 regulating catechin biosynthesis [40], as well as CsMYB6 [41], CsMYB73 [42], and CsDOF [43] involved in theanine biosynthesis regulation. In summary, although there is a degree of understanding of the key flavor substance synthesis genes and key upstream TFs, little is known about the role played by 5mC methylation in the synthesis of key flavor metabolites.

In this study, a global high-throughput multiomics analysis including 5mC methylation sequencing was conducted to investigate the effects of 5mC methylation on tea plant growth and development and pre- and post-harvest flavor substance synthesis. First, we characterized the 5mC methylation differences among four key pre-harvest tissues of tea plant using whole-genome bisulfite sequencing (WGBs-seq). We then combined WGBs-seq and RNA-seq to analyze the effects of 5mC methylation on tissue functional differentiation and theanine synthesis in the tea plant. Finally, we further integrated WGBs-seq, the non-volatile and volatile metabolome, and RNA-seq to identify differential flavor metabolites during processing, and further explored the role of 5mC methylation in regulating the synthesis of important flavor substances during post-harvest processing. Our results confirm the important role of 5mC methylation in tea plant growth and flavor substance formation, and extend the study of key flavor substance synthesis mechanisms to the epigenetic level.

## Results

### 5mC methylation plays an important regulatory role in the functional differentiation of tea plant pre-harvest tissues

**Figure 1:**
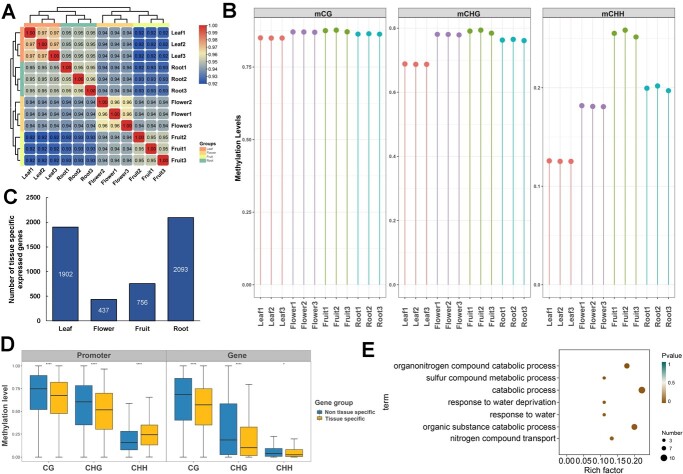
5 mC methylation levels differ across four key pre-harvest tissues of tea plant (**A**) Heat map of Pearson correlation coefficients of WGBS-seq data across tissues. (**B**) Methylation levels of CHH, CHG, and CG across tissues. (**C**) Number of tissue-specifically expressed genes. (**D**) Differences in 5mC methylation levels of tissue-specifically and non-tissue-specifically expressed genes. (**E**) GO annotation of root tissue-specific hypomethylated genes.

Epigenetic modifications are considered to have a significant effect on organ differentiation, and the role of 5mC methylation in animal tissue specificity has been demonstrated [9–11]. However, it is not known whether 5mC methylation also plays an important role in the functional differentiation of plant tissues and whether it is biologically important for the synthesis of tea taste-related substances. We thus performed genome-wide 5mC methylation and transcriptomic analysis of four representative tissues in pre-harvest (root, stem, leaf, flower) of tea plant (*C. sinensis* var. *sinensis* cv. ‘Tieguanyin’). We quantified the 5mC methylation levels of each tissue with a 600-bp length bin, and the correlation analysis (Fig. 1A) and principal component analysis (PCA) (Supplementary Data Fig. S1) of 5mC methylation quantification results showed that the three biological replicates of the same tissue tended to cluster together, and different tissues could be clearly distinguished. mCG, mCHG, and mCHH were all maintained at relatively low 5mC methylation levels in the leaf tissues relative to the other tissues, while all three types of 5mC methylation levels were higher in the fruit tissues relative to the other three tissues. The mCHH 5mC methylation levels of leaf tissues were significantly lower than those of the other three tissues, and the four tissues showed great differences in mCHH 5mC methylation (Fig. 1B). This implies that the tissues have different 5mC methylation statuses.

To investigate whether 5mC methylation would have an effect on tissue-specific expression in pre-harvest processing, we classified all genes into tissue-specifically expressed genes and non-tissue-specifically expressed genes based on TAU values (TAU ≥0.8) and observed the 5mC methylation levels of these two gene sets. A total of 1902, 437, 756, and 2093 tissue-specifically expressed genes were identified in leaves, flowers, fruits, and roots, respectively (Fig. 1C), and the Gene Ontology (GO) annotations of these tissue-specifically expressed genes were also enriched in their own tissue identity-related pathways (Supplementary Data Fig. S2). We found significant differences in 5mC methylation levels between tissue-specifically and non-tissue-specifically expressed genes in all tissues (Fig. 1D, Supplementary Data Fig. S3). Compared with tissue-specifically expressed genes, non-tissue-specifically expressed genes showed higher levels of CG, CHG, and CHH 5mC methylation in the gene body regions and had higher levels of CG and CHG 5mC methylation in the gene promoter regions but had lower levels of CHH 5mC methylation in the gene promoter regions (Fig. 1D). On the other hand, we used roots as test samples to extract genes that are specifically hypomethylated in roots relative to other tissues to check whether these genes were associated with tissue-specific functions. A total of 387 genes specifically hypomethylated in roots were obtained, and the results of KEGG (Kyoto Encyclopedia of Genes and Genomes) annotation of these genes showed that they are indeed closely related to root-specific biological activities, such as response to water and transport of nitrogenous substances (Fig. 1E). These results indicated that differential 5mC methylations among tissues are involved in regulating tissue-specific expression.

### 5mC methylation is involved in regulating dominant theanine synthesis in roots and response to abiotic stresses in tea plant

**Figure 2:**
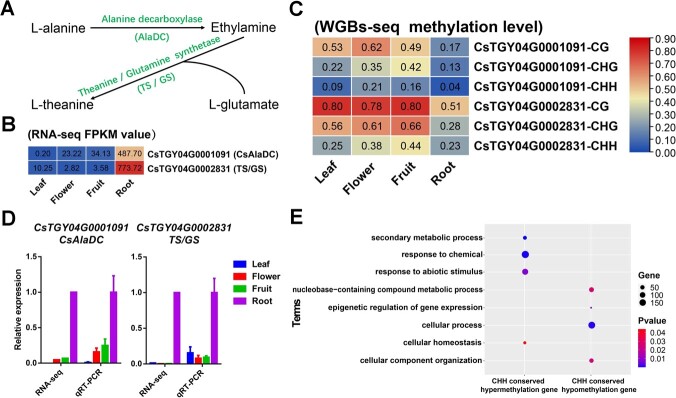
5 mC methylation involved in regulating dominant theanine synthesis in roots and response to abiotic stresses in tea plants. (**A**) Key substrates and genes for theanine synthesis. (**B**) Expression levels of *CsAlaDC* and *TS*/*GS* genes in four tea plant tissues. (**C**) Methylation levels of *CsAlaDC* and *TS*/*GS* genes in four tea plant tissues. (**D**) Relative expression levels of *CsAlaDC* and *TS*/*GS* genes in four tea plant tissues. (**E**) GO annotations of conserved CHH hypermethylated genes and hypomethylated genes.

Previous studies have shown that the important taste substance theanine is mainly synthesized in tea plant roots [44]. However, the epistatic regulatory mechanism of its specific synthesis in roots is not clear. Among the 387 genes specifically hypomethylated in roots, we identified two key genes (*CsAlaDC* and *TS*/*GS*) responsible for theanine synthesis (Fig. 2A), which have lower 5mC methylation levels and higher expression levels in roots relative to other tissues (Fig. 2B–D), which corresponds to their functional characterization in root-dominant theanine synthesis. This result provides epistatic regulatory insight into the tissue-specific synthetic profile of theanine.

In addition, we explored the possible biological significance of conserved hypermethylated and conserved hypomethylated genes in all tissues. We selected the top 2000 genes with the highest 5mC methylation levels and the top 2000 genes with the lowest 5mC methylation levels in each tissue for Venn analysis to search for conserved hypermethylated genes and hypomethylated genes. In total, 612 conserved CG hypermethylated genes, 1152 conserved CHG hypermethylated genes, 1110 conserved CHH hypermethylated genes, 1329 conserved CG hypermethylated genes, 1344 conserved CHG hypermethylated genes, and 364 conserved CHH hypermethylated genes were obtained (Supplementary Data Fig. S4). The GO annotation results showed that the CG and CHG types either conserved hypermethylated genes or hypomethylated genes annotated as basic life activity-related pathways (Supplementary Data Table S1). The conserved CHH hypomethylated genes were annotated as basic life activity-related process pathways, while the conserved CHH hypermethylated genes were related to abiotic stress stimuli, chemical substance responses, and cellular homeostasis (Fig. 2E). Previous studies have reported that changes in 5mC methylation play a non-negligible role in cold stress in tea plants [4, 5]. In rice, CHH 5mC methylation is also demonstrated to play a pivotal role in rice stress adaptation [45]. We therefore hypothesized that CHH 5mC methylation mechanisms may also play an important role in the response to tea plant stresses. The hyper-CHH 5mC methylation status of these stress-related genes under a normal environment may be a mechanism for plant energy saving, while the rapid reduction of 5mC methylation levels of these genes stimulated by stresses can cause upregulation of gene expression levels to adapt to the stress environments.

### Metabolite changes driven by stress during oolong tea post-harvest processing

**Figure 3:**
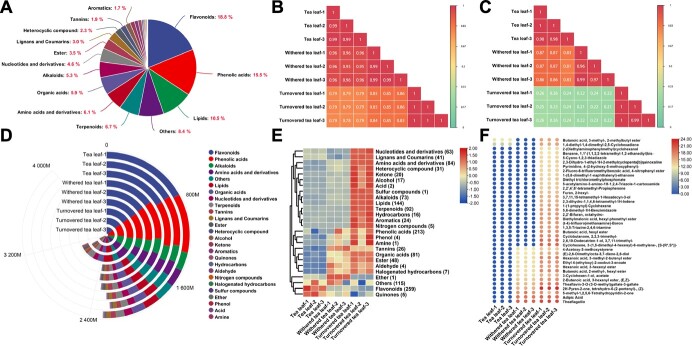
Changes in metabolites during oolong tea post-harvest processing. (**A**) Classification of 1377 secondary metabolites. (**B**) Correlation result of non-volatile metabolite contents detected by UPLC–MS/MS. (**C**) Correlation result of volatile metabolite contents detected by GC–MS. (**D**) Total content changes of all metabolites at three processing points. (**E**) Changes in the content of different classes of metabolites at three processing points. (**F**) Newly emerged metabolites at each processing point.

To identify key flavor substances during post-harvest processing, nine samples were selected for metabolomic analysis at three post-harvest processing points of oolong tea, namely, tea leaf, withered leaf, and turned-over leaf. A total of 1377 metabolites were obtained using UPLC–MS/MS (1147 non-volatile metabolites) and GC–MS (230 volatile metabolites), with the largest proportion being flavonoids, followed by phenolic acids, lipids, terpenoids, amino acids and derivatives, organic acids, and alkaloids, all of which accounted for >5% (Fig. 3A). The correlation analysis revealed that turned-over leaf showed relatively poor correlation with tea leaf and withered leaf, especially in volatile substances (Fig. 3B and C), implying that the metabolites in tea leaves underwent drastic content changes during the turnover process, especially volatile substances.

Since changes in metabolite content and ratios can affect the flavor of tea, we observed changes in total metabolite content and per class content. The content of total metabolites tended to increase during processing (Fig. 3D), which benefited from the simultaneous increase in volatile and non-volatile metabolites (Fig. 3E), especially the 2-fold increase in volatile substances after the turnover process (Supplementary Data Fig. S5). The contents of lipids and terpenoids contributing to tea aroma significantly increased after turnover, indicating that changes in metabolites driven by multiple stresses during turnover are important for enhancing tea aroma (Fig. 3E). Importantly, the whole processing process facilitated the continuous generation of many new secondary metabolites (Fig. 3F), and these newly emerged metabolites contributed to the correlation differences between tea leaf, withered leaf, and turned-over leaf.

### Identification of differential metabolites and differentially expressed genes in oolong tea post-harvest processing

**Figure 4:**
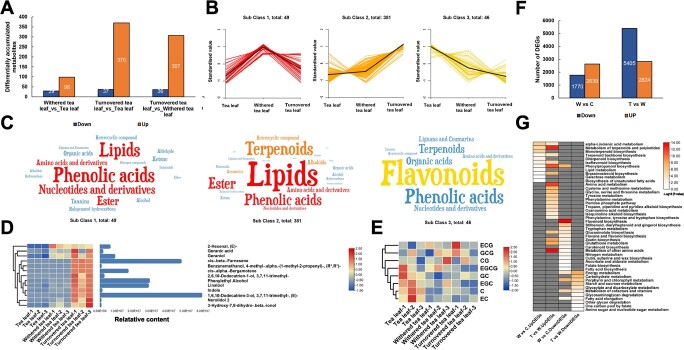
DMs and DEGs in oolong tea post-harvest processing. **(A**) Differential secondary metabolites between different processing points. (**B**) *K*-means clustering of all differential secondary metabolites. (**C**) Word cloud map of each subclass. (**D**) Heat map of changes in contents of essential aroma substances. (**E**) Heat map of changes in contents of important catechins. (**F**) Number of DEGs between different processing points. (**G**) KEGG enrichment pathways of DEGs. C, tea leaf; W, withered tea leaf; T, turned-over tea leaf.

Compared with tea leaf, the majority of metabolites had significant upregulation in content after withering and turnover, and only a few metabolite contents showed a decrease (Fig. 4A). In the three pairwise differential combinations, 127, 407, and 343 differential metabolites (DMs) were obtained, of which 55 were common to the three differential combinations, implying that these 55 metabolites underwent drastic changes during processing (Supplementary Data Fig. S6). All DMs were classified into three subclasses based on content changes (Fig. 4B). Subclass 1 highlighted metabolites that underwent a significant increase in content after withering, including phenolic acids, lipids, nucleotides and derivatives, esters, and amino acids and derivatives (Fig. 4C). After turnover, lipids, terpenoid phenolic acids, ester, amino acids and derivatives, alkaloids and other metabolites were significantly increased, while the content of a large number of flavonoids and some phenolic acids related to bitterness and astringency of tea leaves tended to decrease during the whole of processing (Fig. 4C, Supplementary Data Fig. S7).

We further observed 13 DMs closely related to aroma with clear content changes at three different points and found that the contents of aroma-related metabolites all doubled at the turnover stage, indicating that turnover is the key stage for oolong tea aroma production (Fig. 4D). In contrast, catechins did not show fold-level content changes between different processing points. For instance, the catechins ECG, GCG, CG, and EGCG showed no clear change in content, while GC, EGC, C, and EC had the highest content in tea leaves, and the content showed a small decreasing trend after later withering and turnover (Fig. 4E).

To explore the transcriptional regulatory mechanisms underlying these flavor-related metabolite content changes, we performed RNA-seq of these nine samples. Withering resulted in 2639 upregulated genes and 1770 downregulated genes. Turnover induced the upregulated expression of 2824 genes and the downregulated expression of 5405 genes (Fig. 4F). These differentially expressed genes (DEGs) can effectively explain the content changes of important flavor metabolites during processing. For example, the upregulated DEGs in W (withered tea leaf) versus C (tea leaf) and T (turned-over tea leaf) versus W were significantly enriched in the monoterpene, diterpene, terpene backbone biosynthesis, and terpenoid metabolism-related pathways, which coincided with the significant increase in terpenoids in these two processes (Fig. 4G). Additionally, the fact that upregulated expressed genes were significantly enriched in the phenylpropanoid biosynthesis, isoflavone biosynthesis (this is in a competitive relationship with the flavonoid synthesis pathway), and lipid metabolism pathways corresponded with the decrease in flavonoids and increase in lipid metabolites (Fig. 4G). In addition, the downregulated DEGs were significantly enriched in the flavonoid biosynthesis and flavonoid and flavonol biosynthesis pathways, further explaining why flavonoid, and flavonol biosynthesis was reduced during processing (Fig. 4G).

### Single-base resolution 5mC methylation pattern in oolong tea post-harvest processing

**Figure 5:**
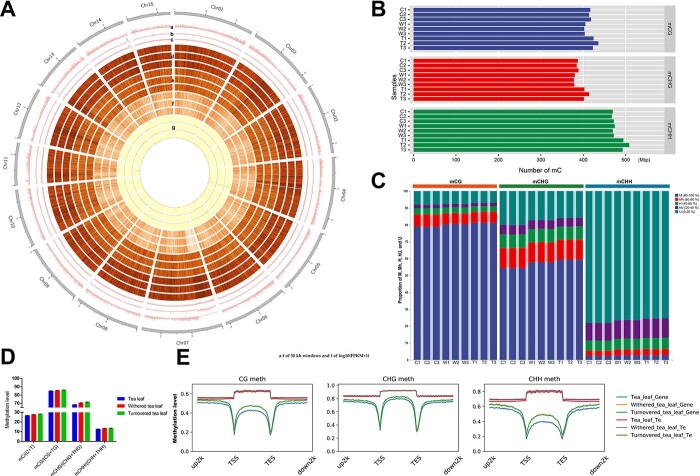
Single-base resolution 5mC DNA methylation profile of three important processing points of oolong tea. (**A**) Circos plot of gene density (a), transposon density (b), GC content (c), CG, CHH, and CHG 5mC methylation levels (d-f), and gene expression levels (g). In d–g, from outside to inside circle: tea leaf, withered leaf, and turned-over leaf. (**B**) Methylation levels of three important processing points. (**C**) Number of methylated cytosines at each processing point. (**D**) Classification of 5mC methylation levels at each processing point. (**E**) Methylation levels of genes and transposable elements at three different processing points. In C and D: C1–C3, tea leaf; W1–W3, withered leaf; T1–T3, turned-over leaf.

To investigate 5mC methylation level changes in tea plant leaves during post-harvest processing, we generated a total of 820.78 Gb of high-quality WGBs-seq data for nine samples, with an average sequencing genome depth of 29.80× per sample (Supplementary Data Table S2). Overall, the gene-enriched genetic regions showed low 5mC methylation levels, while the transposon-rich regions showed high 5mC methylation levels (Fig. 5A), which was consistent with a previous tea plant 5mC methylation study showing that 5mC methylation levels were positively correlated with transposon density [5]. Sample correlation clustering and PCA clearly distinguished the processed leaves (withered leaf and turned-over leaf) from unprocessed fresh leaves (tea leaf) into two groups, and the two processed leaves tended to cluster into one group, implying that processing caused significant changes in genome-wide 5mC methylation levels (Supplementary Data Fig. S8). The 5mC methylation levels of mCG, mCHG, and mCHH in tea leaf were 84.88, 68.41, and 12.60%, respectively, and the 5mC methylation levels of all three types of 5mC methylation were significantly higher after withering and turning over (Fig. 5B). To dissect the reasons for the increase in 5mC methylation levels after withering and turning over, we counted the numbers of methylated cytosines and the 5mC methylation levels of all methylated cytosines (Fig. 5C and D). Withering caused a small decrease in the number of methylated cytosines of the CG and CHG types and a small increase in the number of methylated cytosines of the CHH type, and increased the percentage of high-level methylated cytosines. Differently, turnover caused a significant increase in the number of methylated cytosines of all three types, accompanied by an increase in the percentage of high-level methylated cytosines (Fig. 5C and D). This means that genome-wide 5mC methylation was increased by increasing the 5mC methylation level of some 5mC methylation cytosines during withering, and this process even incurred a small amount of demethylation modification of mCG and mCHG 5mC methylation cytosines. The difference is that, besides the increased 5mC methylation level of the original methylated cytosines during the turnover process, *de novo* 5mC methylation is also important in increasing genome-wide 5mC methylation levels. We also noticed that these two processing processes caused a significant increase in genome-wide 5mC methylation levels both in the body region and in the upstream and downstream regions of genes (Fig. 5E). However, the genome-wide 5mC methylation level changes in transposable elements (TEs) were not significant, except for CHH-type 5mC methylation, which occurred with a small increase in the upstream and downstream regions of TEs. This implies that oolong tea processing mainly regulates gene expression changes by altering gene 5mC methylation levels rather than altering TE 5mC methylation levels.

### 5mC methylation is involved in epistatic regulation of flavor substance-related gene expression in post-harvest processing

**Figure 6:**
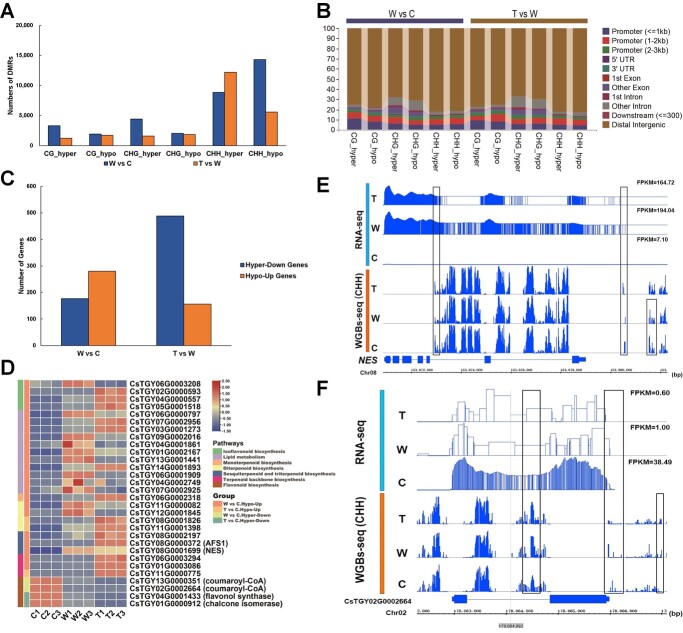
DMRs and DMR-mediated DEGs during oolong tea processing. (**A**) Number of DMRs. (**B**) Location distribution of DMRs in the genome. (**C**) Number of DMR-mediated DEGs. (**D**) Heat map of gene expression levels of DMR-mediated DEGs associated with important flavor substances. (**E**) IGV (Integrative Genomics Viewer) visualization of WGBs-seq (CHH) and RNA-seq data of the *nerolidol synthase* (*NES*) gene. (**F**) IGV visualization of WGBs-seq (CHH) and RNA-seq data of the *4-coumaroyl-CoA* (*CsTGY13G0000351*) gene. C, tea leaf; W, withered tea leaf; T, turned-over tea leaf.

To further investigate the genes that undergo differential 5mC methylation during processing and their potential impact on flavor substance changes, we extracted 120 721 370 5mC methylation cytosines coexisting in all samples for differentially methylated region (DMR) identification using MethylKit [46], HOME [47], and batDMR [48], and differentially methylated promoter (DMP) identification using MethylKit. A total of 15 836 hypermethylated DMRs (Hyper-DMRs), 19 047 hypomethylated DMRs (Hypo-DMRs), 146 Hyper-DMPs, and 75 Hypo-DMPs were obtained in W versus C, and a total of 15 662 Hyper-DMRs, 25 897 Hypo-DMRs, 62 Hyper-DMPs, and 68 Hypo-DMPs were obtained in T versus W (Fig. 6A). ChIPseeker annotation of DMRs showed that CG-type DMRs had a higher percentage of distribution in the promoter region, especially within the upstream 1-kb promoter region of the gene (>8.26%), than the other two types (Fig. 6B). However, CHG-type DMRs had a higher frequency of distribution in the other exon and other intron regions (3.91–11.11%), and ~81% of CHH-type DMRs fell in the distal intergenic region, much higher than ~76% of CG-type and ~69% of CHG-type DMRs (Fig. 6B). This implies that different types of 5mC methylation play a biased role in 5mC methylation in different regions of the gene.

We considered genes with overlap with DMRs in the gene body region or 1.5 kb upstream or 0.5 kb downstream of the gene as genes potentially affected by 5mC methylation (Supplementary Data Fig. S9) and further screened DMR-mediated DEGs based on differential gene expression (|log_2_fold change| ≥ 1.5 and *P*-value ≤.05) (Fig. 6C). A total of 259 hypermethylation-mediated downregulated genes (Hyper-Down genes) and 389 hypomethylation-mediated upregulated genes (Hypo-Up genes) were obtained in W versus C; 563 Hyper-Down genes and 198 Hypo-Up genes were obtained in T versus W (Fig. 6C). The KEGG annotations showed that these DMR-mediated DEGs were responsible for lipid metabolism, terpenoid synthesis, and flavonoid/isoflavonoid biosynthesis, implying that 5mC methylation plays critical roles in the changes in flavor substance content during processing (Fig. 6D). For example, the increase in aroma-related terpenoids during processing benefited from upregulated gene expression levels mediated by hypomethylation modifications of terpenoid synthetic genes during processing, including well-known genes, namely, the *NES* (*nerolidol synthase*) gene important for nerolidol synthesis (Fig. 6E) and the *AFS1* (*α-farnesene synthase*) gene for α-farnesene synthesis (Supplementary Data Fig. S10). We found that four isoflavonoid-related synthetic genes were hypomethylated during processing accompanied by upregulated gene expression, while four flavonoid-related synthetic genes underwent the exact opposite changes, which included the *CsTGY02G0002664* and *CsTGY13G0000351* genes encoding two 4-coumaroyl-CoAs, the important rate-limiting enzymes in the flavonoid synthesis pathway (Fig. 6D and F, Supplementary Data Fig. S10). In addition, the upregulated expression of seven lipid metabolism-related genes mediated by hypomethylation coincided with increased lipid contents (Fig. 6D). To confirm the accuracy of gene expression changes from RNA-seq, we further verified the expression of six of the important metabolite-related genes by qRT–PCR (Supplementary Data Fig. S11). These findings suggested that methylation-mediated gene expression plays an important role in the production of aroma and taste substances during oolong tea processing.

## Discussion

In plants, 5mC methylation is closely related to more than one biotic or abiotic stress and several secondary metabolite syntheses [4, 5, 7]. However, studies on 5mC methylation in the tea plant are very limited, only involvingcold stress [4, 5], as well as ABA and indole accumulation [6, 7], during tea post-harvest processing. These several important studies imply that 5mC methylation plays an important upstream regulatory role in both pre-harvest and post-harvest processing in the tea plant, and also suggest that 5mC methylation in tea trees may be involved in more than these regulations. Recently, high-throughput WGBs-seq, RNA-seq, and metabolome technologies have provided us with the opportunity for comprehensive and high-throughput investigation of the potential impact of 5mC DNA methylation on tea plant growth and flavor metabolite synthesis. Our results demonstrated that 5mC DNA methylation is not only involved in cold stress and the synthesis of a small number of metabolites [4–7], but also plays significant roles in the functional differentiation of tea plant tissues and in the synthesis of important flavor substances (e.g. theanine, catechins, and terpenoids) in both pre- and post-harvest processing. The differentiation of the same stem cell into different tissues is similar to the process undergone by tissue cancers; these results suggest that differences in 5mC methylation are closely related to functional differentiation of tissues, and this close correlation has been demonstrated in animal studies [9–11]. We also found significant differences in 5mC methylation levels among tea plant pre-harvest tissues, especially for CHH-type 5mC methylation. Combined with gene expression analysis, we found that 5mC methylation was closely related to the functional differentiation of tea plant tissues, and genes related to tissue characteristic life activity maintained relatively low 5mC methylation levels, so as to be responsible for the dominant expression level of these tissue characteristic genes. This implies that, similar to animals, 5mC methylation regulation in the tea plant also plays an important role in the functional differentiation of tea plant pre-harvest tissues.

Theanine is a non-protein amino acid unique to the tea plant, which gives the tea its ‘freshness’ [13]. Currently, extensive studies have shown that the *CsAlaDC* and *CsTSI* genes synergistically determine the high synthesis of theanine in the tea plant [49], and that CsMYB40 and CsHHO3 act as upstream regulatory factors to regulate the gene expression level of the *CsAlaDC* gene [50]. However, the activity of theanine synthesis in different tissues of the tea plant varies greatly, and theanine biosynthesis occurs mainly in the roots [44, 51]. The regulatory mechanism of this biased distribution is not clear. The results of expression analysis of different tissues showed that the expression level of the *CsTSI* gene is positively correlated with the theanine content among different tissues of the tea plant [51, 52]. The present study provides a possible regulatory mechanism for this bias from the perspective of 5mC methylation. We found that the dominant synthesis of theanine in tea plant roots is strictly controlled by 5mC methylation regulatory mechanisms. Among tissues, the level of theanine content was negatively correlated with the 5mC methylation level of two key theanine synthesis genes, which suggested that we may manipulate the 5mC methylation level of theanine synthesis genes to achieve high throughput of theanine synthesis in whole tissues or in leaf tissues of the tea plant. This has important implications for the precise manipulation of tea flavor, especially when gene editing tools are mature.

Studies on cold stress in the tea plant have demonstrated that the level of 5mC methylation is altered under cold stress and that changes in the gene expression of stress response-related genes are closely associated with altered 5mC methylation levels [4, 5]. Studies on some other crops have also confirmed the important role of CHH 5mC methylation in various abiotic stress responses and adaptations [45, 53, 54]. This study found that CHH conserved hypermethylated genes were closely associated with abiotic stress responses under normal survival conditions, suggesting that hypermethylation modification of stress-responsive genes under a normal environment may have been the mechanism for plant energy saving, and would initiate demethylation, thus causing upregulation of gene expression of stress-responsive genes only under an adverse environment. This novel finding implies that CHH 5mC methylation also plays a key regulatory role in initiating abiotic stress responses.

The unique aroma of the finished oolong tea of ‘Tieguanyin’ is not only related to the amount of aroma substance precursors in the pre-harvest fresh leaves but is also largely due to its unique post-harvest processing. We found that the aroma-related substances of oolong tea substantially increased during processing, especially well-known substances such as α-farnesene and nerolidol, which further confirmed the importance of the processing process in endowing the finished tea with a unique flavor [32–35]. Although some studies have now shown that the high synthesis of these important aroma substances during processing is due to the significant upregulation of their synthetic genes during processing [7, 32–35], knowledge of the upstream regulatory mechanisms of these key genes is limited, especially with regard to epistatic regulatory mechanisms, because the tea plant is a non-model plant without a transformation system. This study revealed that the weighted 5mC methylation level is elevated during oolong tea post-harvest processing. Based on multiomics results, we further evidenced that 5 mC methylation changes can ultimately affect the accumulation of flavor substances by regulating the expression level of key flavor synthesis genes. In the present study, aroma-related terpenoids such as α-farnesene, nerolidol, and taste-related flavonoids containing various catechins were also demonstrated to be strictly regulated by 5mC methylation, similar to what has been previously reported for ABA and indole [6, 7]. We also found that a large number of lipid-related synthetic pathway genes were affected by 5mC methylation during post-harvest processing, which in turn upregulated their gene expression levels to increase the aroma of processed tea. This study extends the study of flavor substances in oolong tea processing to the level of epigenetic regulation, which is important for enhancing the quality of tea leaves.

However, the reason for elevated methylation during processing needs to be further explored in future. In *Arabidopsis*, MET1 (METHYLTRANSFERASE 1), CMT3 (CHROMOMETHYLASE 3), and CMT2 (CHROMOMETHYLASE 3) are respectively responsible for the maintenance of 5mC DNA methylation in the CG, CHG, and CHH contexts, and in the CG, CHG, and CHH contexts DNA can be 5mC­methylated *de novo* by domain­rearranged methyltransferases (DRMs) through the RNA-directed DNA methylation (RdDM) pathway [55–57]. In recent years, several enzymes related to 5mC demethylation, including DME (DEMETER), DML (DEMETER-LIKE), and ROS1 (REPRESSOR OF SILENCING1), have been reported [58]. In previous studies on the dynamics of the 5mC methylation level during fruit development and ripening in sweet orange and tomato, alterations in 5mC methylation levels were considered to be closely related to changes in gene expression of DNA methylase or demethylase genes [59, 60]. For instance, the increase in DNA methylation levels in sweet orange was attributed to the continuous decrease in the expression levels of four DNA demethylase-related genes (*CsDME*, *CsDML1*, *CsDML3*, and *CsDML4*) during ripening [59]. Besides, the decrease in methylation levels during tomato ripening was mainly a result of the continuous downregulation of several RNA-directed DNA methylase-related genes, including *FvDRM1.3* and *FvDRM3.1* [60]. Compared with the long-time-scale change process of fruit ripening [59, 60], the elevated weighted methylation level during tea processing could not be fully and effectively explained by the expression upregulation of DNA methylase or expression downregulation of the demethylase gene, which might suggest that the mechanism of methylation changes in short-time-scale processing might be more complex. We found that most DNA methylases and demethylase genes showed a very low FPKM (fragments per kilobase of transcript per million mapped reads) value (<5), and non-significant changes during processing process (Supplementary Data Table S3). Since unavoidable mapping mismatches would result in some non-expressed genes that have a low FPKM value, we thus hypothesized that most DNA methylases and demethylase genes do not play important functions in DNA methylation level changes during oolong tea post-harvest processing. Notably, *CsDRM1* (FPKM >5), may contribute to the increased methylation levels of CG, CHG, and CHH content during the turnover process. But this gene was not significantly upregulated in expression level during the withering process as expected, so it may not contribute to the elevated methylation level during the withering process. The above results imply that other rapid methylation regulatory mechanisms may exist during short-time-scale change processes, such as chromatin alterations and histone modification. As previously reported, CHH is strongly associated with H3K9me2 [57].

## Materials and methods

### Plant material

Tea leaves were collected on 29 April 2021 from the tea plant farm of Anxi County (25.05°N, 118.18°E), Quanzhou City, Fujian Province. The national variety ‘Tieguanyin’, which is suitable for oolong tea, was used as the material for this study, and the leaf collection standard was one bud and two leaves or three leaves. All fresh leaves were harvested at ~4 p.m.

The fresh leaves after harvesting were first withered in sunlight for 35 min and then transferred to indoor withering for 15 min. After withering, the tea leaves were turned over three times using a shaking machine (6CYQT-90, Fujian, China) at 25 r/min: the first turnover process was performed for 5 min, and then the tea leaves were placed indoors for 90 min; the second turnover process was performed for 5 min, and then the tea leaves were placed indoors for 150 min; the third turnover process was performed for 20 min, and then the tea leaves were placed indoors for 180 min.

To study the effects of withering and turnover on metabolites, gene expression, and gene 5mC DNA methylation levels of tea leaves, 100-g samples were randomly collected as three treatment samples, namely, fresh, withered, and turned-over tea leaves, and three independent biological replicates were performed for each treatment. In total, nine samples were collected for metabolome, RNA-seq, and WGBs-seq sequencing.

### Chemicals and reagents

The standards and reagents were purchased from Merck (Darmstadt, Germany), Sinopharm (Shanghai, China), and Sigma (St Louis, MO, USA) unless otherwise noted.

### Determination of non-volatile metabolite components and content

The sample extraction and metabolite profiling analysis using an LC–ESI–MS/MS system (UPLC, Shimadzu Nexera X2; MS, Applied Biosystems 4500 QTRAP) was the same as the method described in a previous paper [61], and all experiments were performed by MetWare (Wuhan, China). Names and properties of metabolites were identified by matching fragmentation patterns, retention times, and accurate *m*/*z* values of metabolites with standards in the MetWare database (MWDB), MassBank, HMDB, and Metlin public databases.

### Determination of volatile metabolite components and contents

The separation and concentration of volatiles and GC–MS conditions were the same as those previously described for *Peganum harmala* L. by Wang *et al*. [62], and all experiments were also performed by MetWare (Wuhan, China). The detection of volatile metabolites by comparison of mass spectra with the MWGC or NIST data system library was achieved, the linear retention index was determined and quantitative analysis was performed using MassHunter software (Agilent, CA, USA).

### Differential metabolite identification and statistical analysis

In pairwise DM analysis, significantly different metabolites were required to meet a fold change ≥2 or ≤0.5, a *P*-value ≤.05, and variable importance in project (VIP) ≥1 [63].

The Pearson correlation coefficient matrix between different samples was measured by the R package ‘cor’ function and visualized by the ‘pheatmap’ package. To study the content changes of metabolites during oolong tea processing, the relative contents of all DMs were normalized by *z*-score, and then all DMs were grouped based on *K*-means clustering results. All heat maps in this study were generated by TBtools software [64].

### Transcriptome sequencing and data analysis

All RNAs of tea plant samples were extracted using the Tiangen Total RNA Extraction Kit (DP441, Beijing, China), followed by mRNA enrichment using magnetic beads with oligo(dT). All cDNAs were reverse-transcribed, sequencing libraries were constructed using the QIAquick PCR Kit (Qiagen, Venlo, The Netherlands), and all sequencing libraries were sequenced on the Illumina HiSeq2500 platform using the paired-end 150 bp method. The raw data were filtered using fastp software with default parameters [65]. The recently published ‘Tieguanyin’ high-quality genome [66] from our laboratory was used as the reference genome, and HISAT2 was used to build the reference genome index and map clean data to the reference genome [67]. The reads were quantified using featureCounts [68], and the genome-wide DEG analysis was performed using DEseq2 [69]. Genes with significant differences in expression levels, |log_2_fold change| ≥ 1.5 and *P*-value ≤.05 were considered DEGs [69] and were then enriched to GO terms and KEGG pathways using TBtools [64].

To screen for tissue-specific expression genes, we first filtered for low-expressed genes: if a gene had FPKM ≥5.0 in any tissue [70] the gene was retained, and uncharacterized genes were filtered. Then, the TAU value of each gene was calculated by the Tissue Specificity Index (TAU) Calc tool of TBtools [64] based on the expression level of the gene in each tissue, and the genes with a TAU value >0.8 were set as the tissue-specific expression gene screening criteria.

### Genome-wide DNA 5mC methylation sequencing and data analysis

The total DNA of all tea plant samples was extracted using a modified CTAB method [71] and analyzed for DNA degradation and RNA contamination using 1% agarose gel electrophoresis; OD260/280 ratios and DNA concentration of all samples were measured separately with Nanodrop and Qubit 2.0. The samples that passed the quality control were selected for subsequent library construction.

A percentage of λ DNA was mixed into the tested DNA samples for assessing the quality of bisulfite conversion rate. Genomic DNA was randomly cut into to 200- to 400-bp DNA fragments using Covaris M220, and the interrupted DNA fragments were end-repaired, A-tailed, and ligated to the sequencing linker in which all cytosines were methylated. After bisulfite treatment (using EZ DNA Methylation Gold Kit, Zymo Research), the unmethylated-C nucleobase becomes T nucleobase after PCR amplification, but the methylated-C nucleobase remains unchanged; finally, PCR amplification was conducted to yield the final WGB libraries. All WGB libraries were sequenced by the paired-end 150 bp method on the Illumina HiSeq sequencing platform.

The WGBs-seq raw data were filtered using fastp [41] with the following parameters: -5 --cut_front_window_size 4 --cut_front_mean_quality 20 -3 --cut_tail_window_size 4 --cut_tail_mean_quality 20 --cut_right --cut_right_window_size 4 --cut_right_mean_quality 20 --detect_adapter_for_pe -q 15 -u 40 -e 20 -n 5 -l 30 -p -P 20 -w 4. We next used BatMeth2 pipel [48] (default parameters) to map the filtered WGB reads to the ‘Tieguanyin’ genome and completed the identification of 5mC methylation sites and calculation of 5mC DNA methylation levels. We further obtained coexisting 5mC methylation sites in all samples and performed the identification of DMRs by MethylKit [46] (parameters: —window 1000 —step 500 —mincov 4), HOME [47] (HOME-pairwise, −d 0.2 -mc 5), and batDMR [48] (−methdiff 0.2 -minstep 100 -mindmc 4 -pvalue 0.05). In addition, the identification of DMPs was performed using MethylKit (—window 600 —step 200 —mincov 4).

To characterize the 5mC methylation level of each gene, we counted the number of methylated cytosines and unmethylated cytosines in the body region of each gene, and the total number of all methylated cytosines as a percentage of the total number of all cytosines was the 5mC methylation level of that gene. When the 5mC methylation level of the gene in one tissue was significantly lower than that in the other three remaining tissues and the difference was >0.15, with significant differences (Student’s test *P*-value ≤.05), the gene was considered to be specifically hypermethylated in that tissue. In addition, we obtained the top 2000 genes with the highest 5mC methylation level and the top 2000 genes with the lowest 5mC methylation level in each tissue separately for Venn analysis, and the two intersecting gene sets obtained from four tissues were used as tissue-conserved hypermethylated gene sets and conserved hypomethylated gene sets, respectively.

### Genome-wide identification of DNA methylase and demethylase

The protein sequences of DNA methylase and demethylase in *Arabidopsis* and *C. sinensis* (var. *sinensis* cv. ‘LJ43’) were obtained from the *Arabidopsis* Genome Database (https://www.arabidopsis.org) and previous publications by Wang *et al*. [4] and Tong *et al*. [5]. Using these sequences as query sequences, we identified DNA methylase and demethylase candidate sequences in the ‘Tieguanyin’ genome using the BlastP function (E-value <E−20) and further filtered these candidate sequences based on the analysis of conserved motifs from SMART (http://smart.embl-heidelberg.de/) and Pfam (http://pfam.xfam.org/search/sequence).

### Quantitative real-time PCR assays

To verify the accuracy of the RNA-seq analysis results, several important metabolite-related genes were extracted by qRT–PCR (primers are listed in Supplementary Data Table S4). Total tea plant RNA was acquired using the FastPure Universal Plant Total RNA Isolation Kit (RC411-01) (Vazyme Biotech Co., Ltd, Nanjing, China) and reversed into cDNA using the PrimeScript RT Reagent Kit (RR037A) (Takara, Japan). The 10-μl qRT–PCR reaction was prepared using the 2x SYBR Green qPCR Master Mix (US Everbright^®^ Inc., Suzhou, China). All qRT–PCRs were conducted on a CFX96 Touch™ Real-Time PCR Detection System (Bio-Rad, Hercules, CA, USA). The tea plant *Actin* and *GAPDH* genes were utilized as internal controls among different samples for qRT–PCR analysis. The 2^−ΔΔCT^ method was used to calculate gene expression fold changes from three independent biological replicates.

## Acknowledgements

This study was funded by Shenzhen Science and Technology Program (Grant No. RCYX20210706092103024) and the Key-Area Research and Development Program of Guangdong Province (2020B020220004). We are grateful to Chinese tea masters Lianggu Chen and Zhipeng Chen for their help in sample collection and oolong tea processing. We thank the anonymous reviewers for helpful comments on this manuscript.

## Author contributions

X.Z. and W.K. conceived the ideas for this paper. W.K. performed all of the experiments, analyzed the data, prepared the figures and tables, and wrote the paper. W.K., Q.Z. (Qiufang Zhu), Q.Z. (Qing Zhang), and J.L. collected or provided plant materials. Y.Z. and J.Y. performed qRT–PCR experiments. K.C., W.L., and M.J. completed the visualization of some of the results. S.Z. assisted in the bioinformatics problems. All authors read and approved the final manuscript.

## Data availability

All sequencing datasets have been deposited in the National Genomics Data Center (NGDC) under accession number PRJCA014523.

## Conflict of interest

The authors declare that they have no competing interests.

## Supplementary data

Supplementary data are available at *Horticulture Research* online.

## Supplementary Material

Web_Material_uhad126
