# Supplementary material for: 5mC DNA methylation modification-mediated regulation in tissue functional differentiation and important flavor substance synthesis of tea plant (Camellia sinensis L.)
Source: Hortic Res. 2023 Jun 13;10(8):uhad126. doi: 10.1093/hr/uhad126 (PMC10407603; doi:10.1093/hr/uhad126)
Supplement: Web_Material_uhad126 [file web_material_uhad126.zip › Fig S.docx]

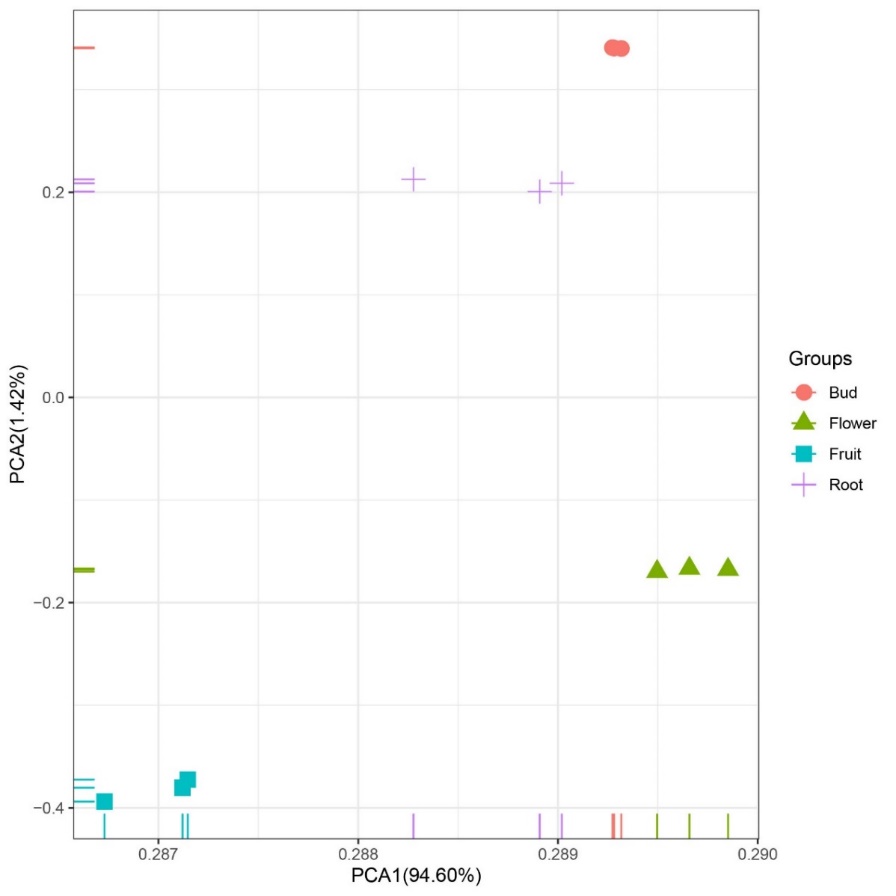


**Figure S1.** Principal component analysis of 5mC DNA methylation levels in different tissues of tea plant


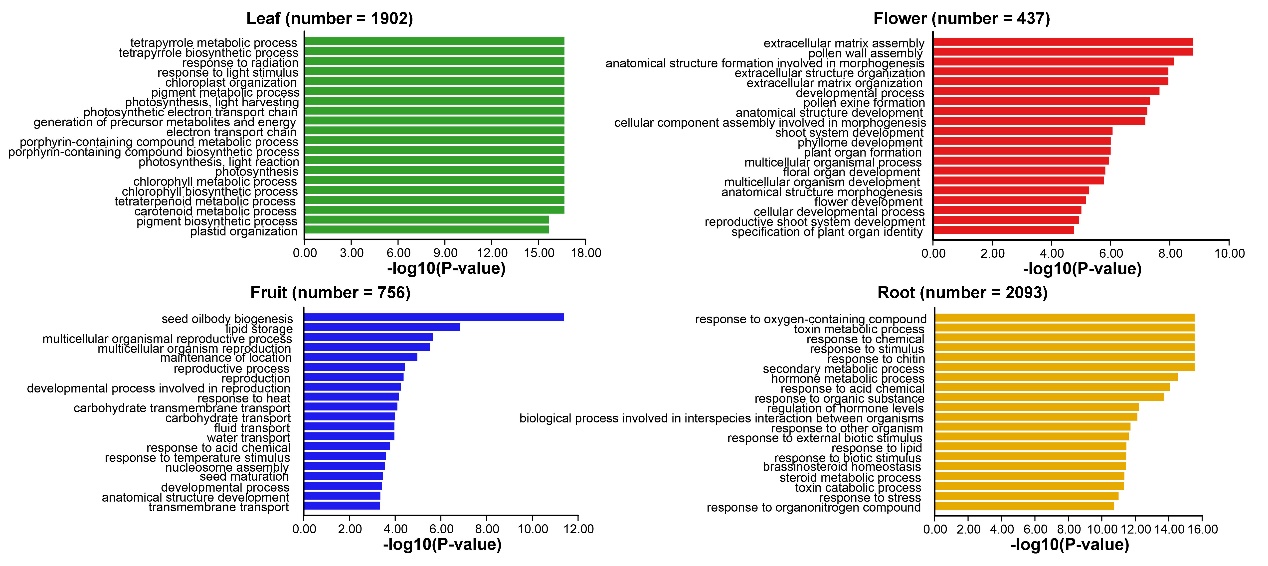


**Figure S2.** GO annotations of tea plant tissue-specific expression genes (P-value ≤ 0.05)


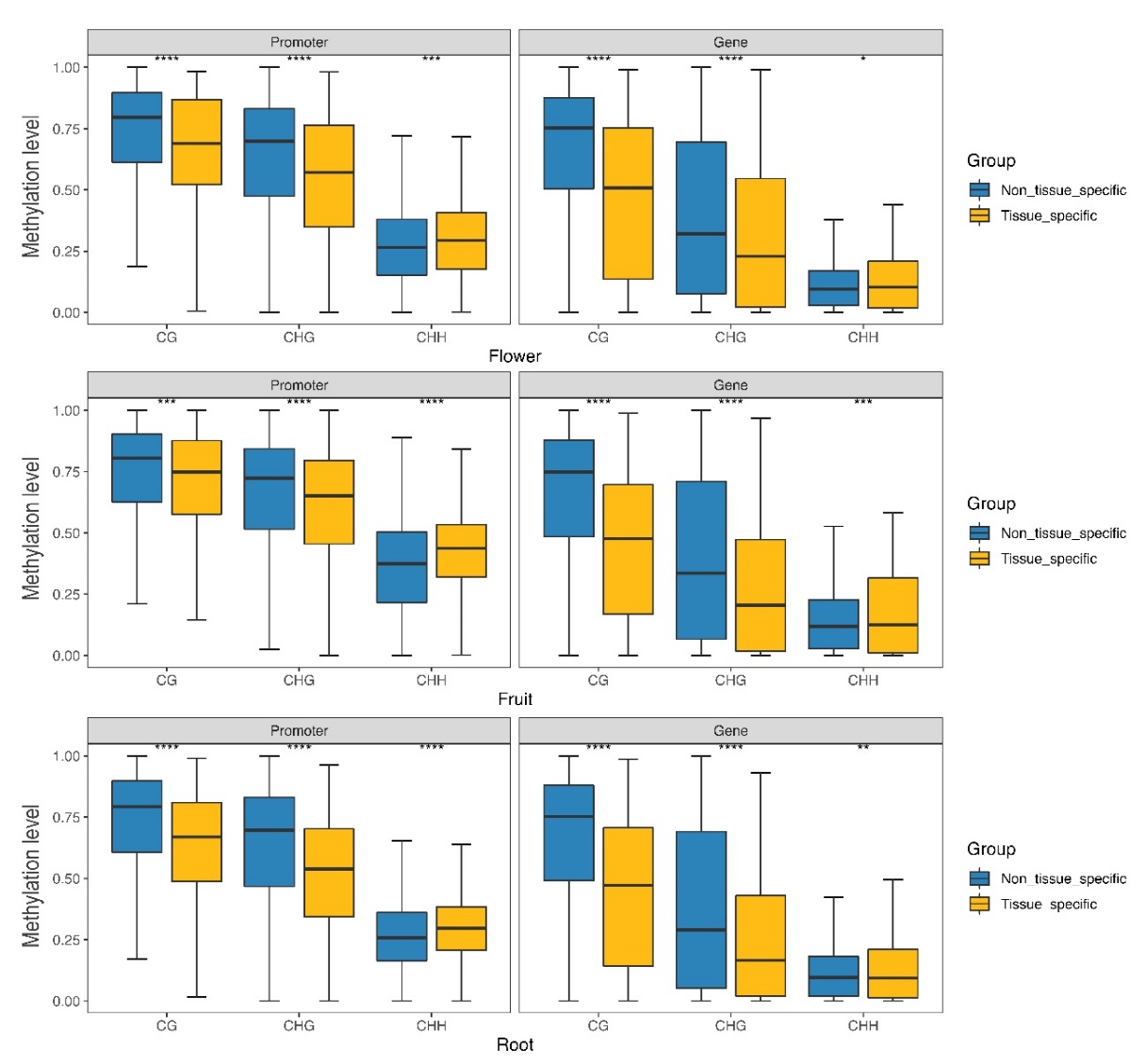


**Figure S3.** Box plots of tissue-specific and non-tissue-specific gene 5mC DNA methylation levels in flower, fruit and root tissues


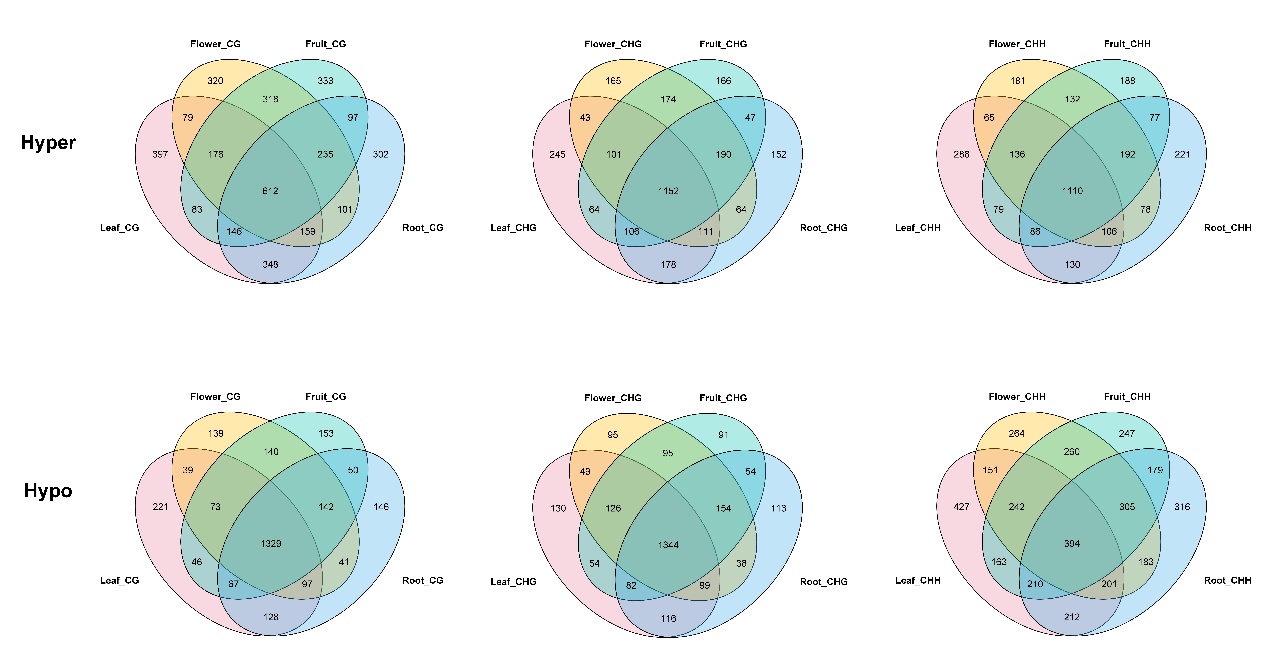


**Figure S4.** Venn analysis of the top 2000 hypermethylated and top 2000 hypomethylated genes in four tissues of tea plant


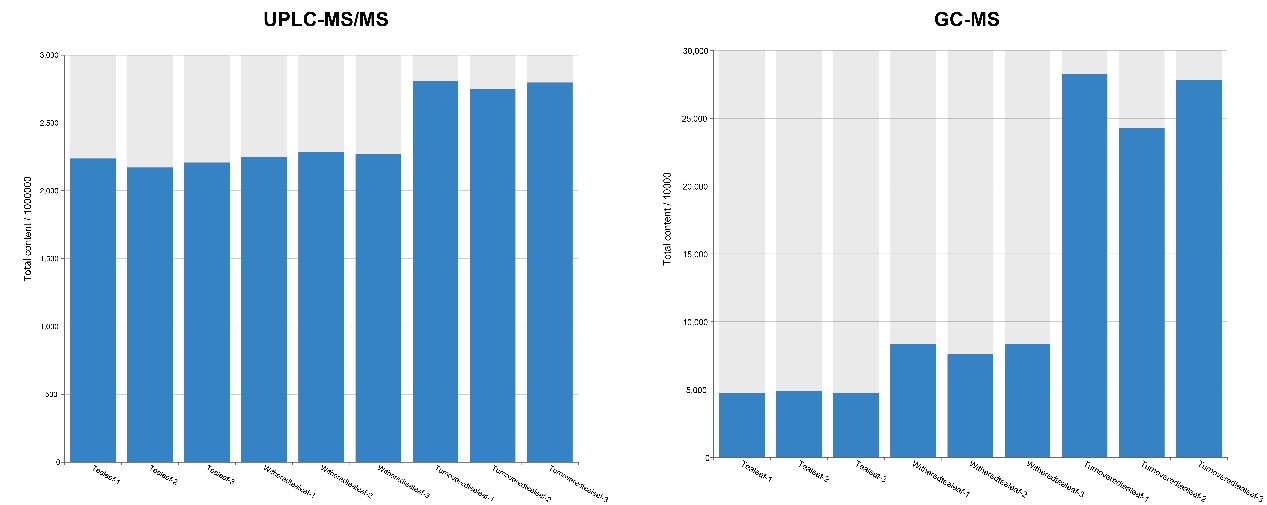


**Figure S5.** Changes in the content of nonvolatile metabolites (UPLC‒MS/MS) and volatile metabolites (GC‒MS) during processing


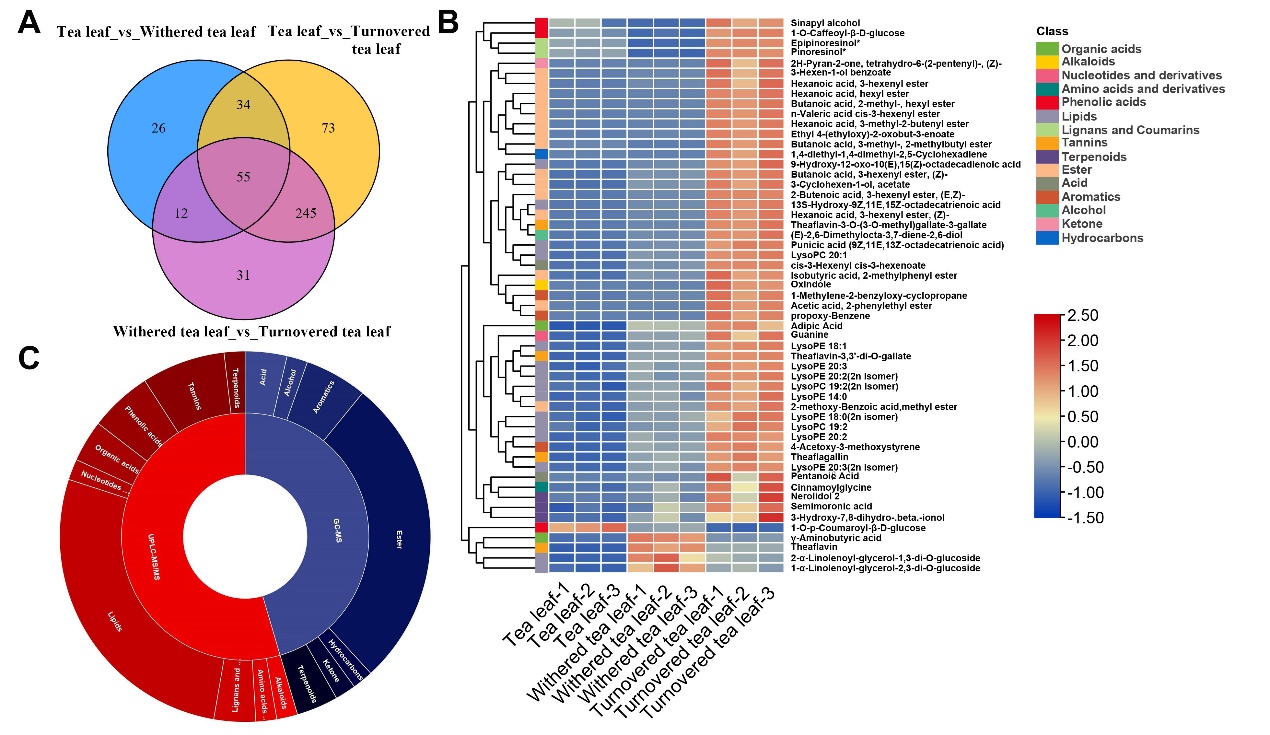


**Figure S6.** Fifty-five differential metabolites coexisting in three differential combinations


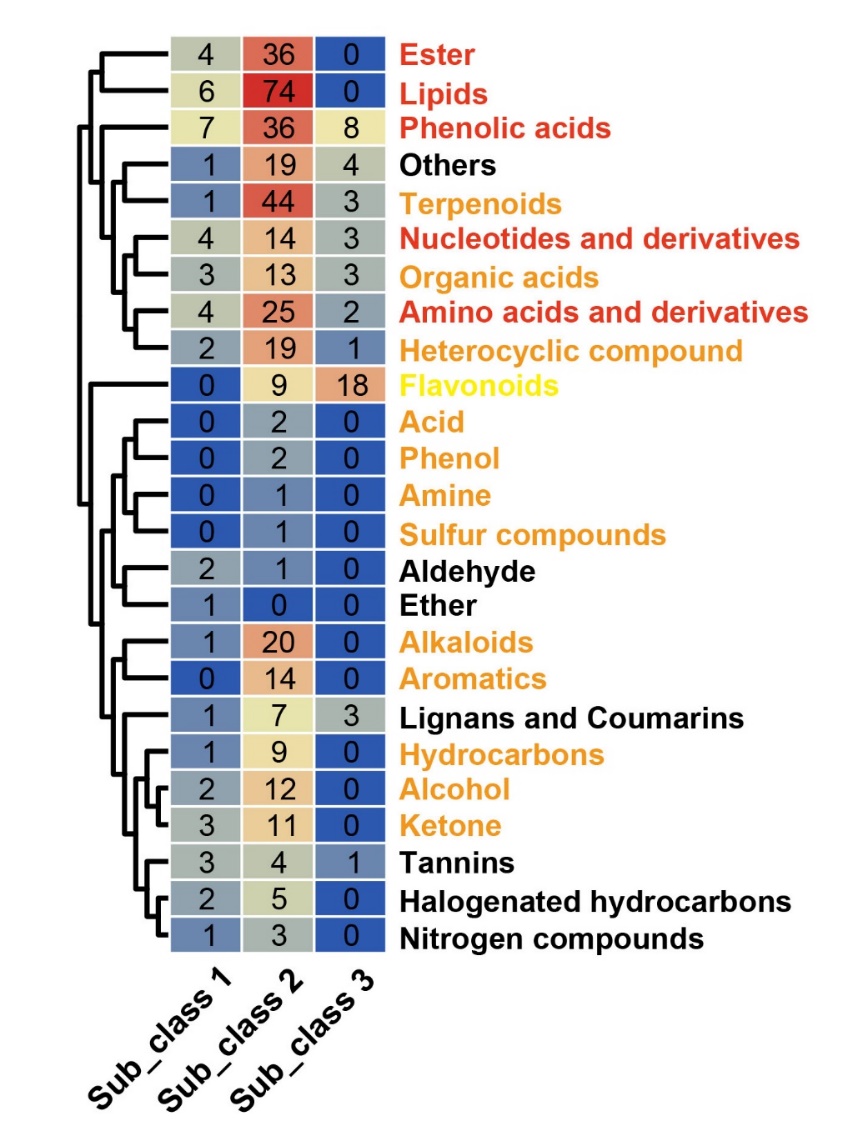


**Figure S7.** Metabolite classification for each Sub_class
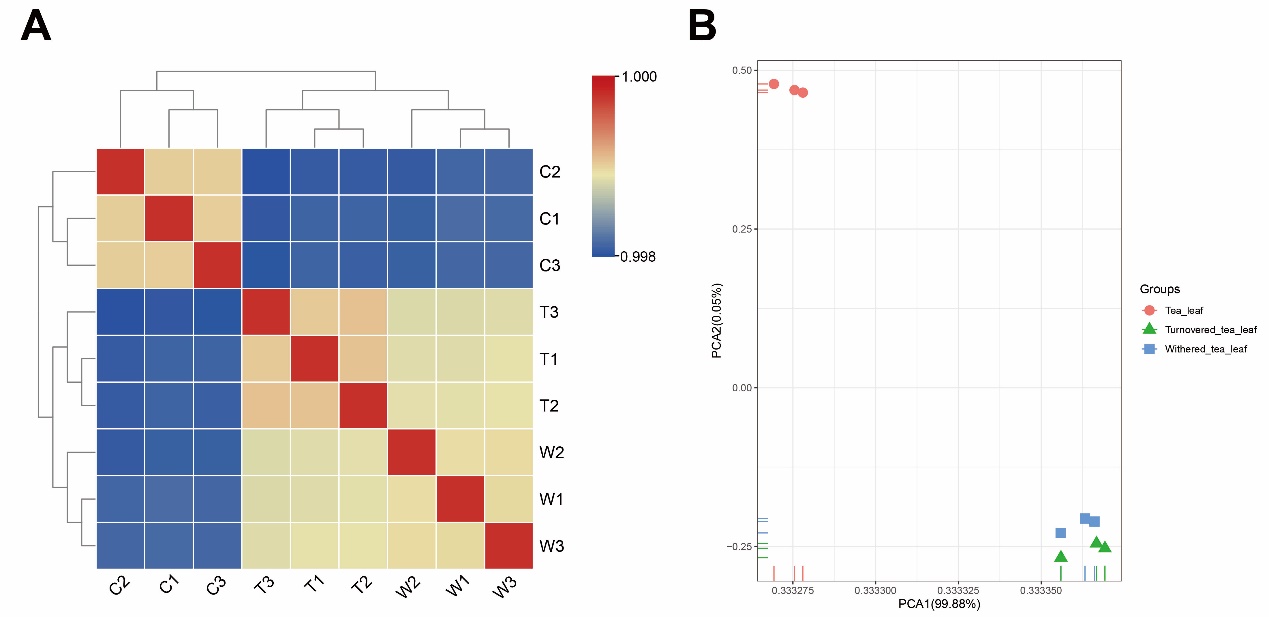


**Figure S8.** Heatmap clustering (A) and the principal component result (B) of 5mC DNA methylation levels of all samples

**Figure S9.** Differentially methylated region-related genes


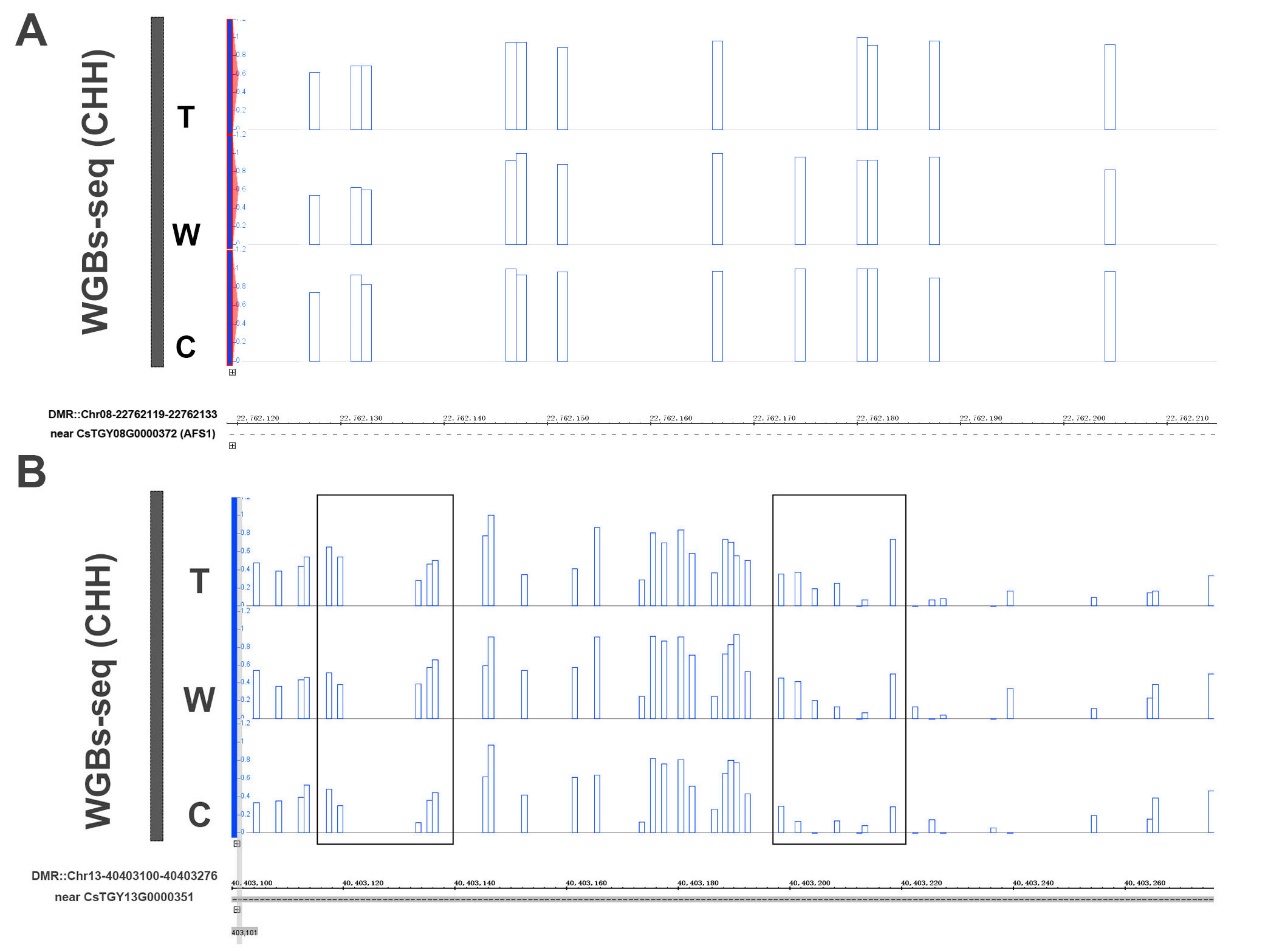


**Figure S10.** *CsTGY08G0000372* and *CsTGY13G0000351* genes near differentially methylated regions


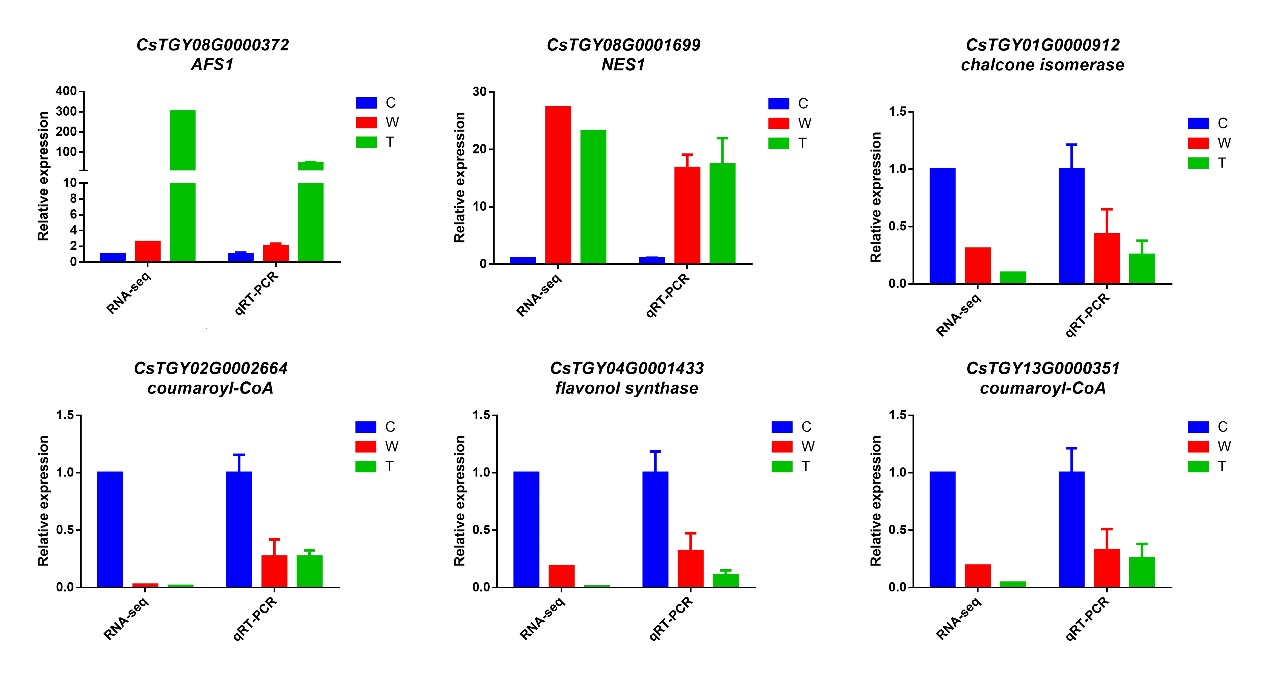


**Figure S11.** qRT-PCR results of six of the important metabolite-related genes
